# A Systematic Review of Structural and Functional MRI Studies Investigating Social Networking Site Use

**DOI:** 10.3390/brainsci13050787

**Published:** 2023-05-11

**Authors:** Michael Wadsley, Niklas Ihssen

**Affiliations:** Department of Psychology, Durham University, Durham DH1 3LE, UK; niklas.ihssen@durham.ac.uk

**Keywords:** social networking sites, social media, addiction, neuroimaging, MRI, systematic review

## Abstract

An understanding of the neurocognitive profile underlying the use of social networking sites (SNSs) can help inform decisions about the classification of problematic SNS use as an addictive disorder and elucidate how/when ‘SNS addiction’ might develop. The present review aimed to synthesize structural and functional MRI research investigating problematic/compulsive forms of SNS use or regular (non-addicted) SNS use behaviours. We conducted a systematic search for research articles published in English using the *Web of Science*, *PubMed*, and *Scopus* databases up to October 2022. Studies meeting our inclusion criteria were assessed for quality and a narrative synthesis of the results was conducted. Twenty-eight relevant articles were identified comprising structural MRI (*n* = 9), resting-state fMRI (*n* = 6) and task-based fMRI studies (*n* = 13). Current evidence suggests that problematic SNS use might be characterised by (1) reduced volume of the ventral striatum, amygdala, subgenual anterior cingulate cortex, orbitofrontal cortex and posterior insula; (2) increased ventral striatum and precuneus activity in response to SNS cues; (3) abnormal functional connectivity involving the dorsal attention network; (4) inter-hemispheric communication deficits. Regular SNS use behaviours appear to recruit regions involved in the mentalising network, the self-referential cognition network, the salience network, the reward network and the default mode network. Such findings are at least partially consistent with observations from the substance addiction literature and provide some provisional support for the addictive potential of SNSs. Nonetheless, the present review is limited by the small number of eligible studies and large heterogeneity in the methods employed, and so our conclusions should remain tentative. Moreover, there is a lack of longitudinal evidence suggesting SNSs *cause* neuroadaptations and thus conclusions that problematic SNS use represents a disease process akin to substance use addictions are premature. More well-powered longitudinal research is needed to establish the neural consequences of excessive and problematic SNS use.

## 1. Introduction

The use of social networking sites (SNSs) is a ubiquitously popular activity that has seen a surge over the last decade. In 2012, it was estimated that there were approximately 1.5 billion SNS users [1]. In 2022, the number of SNS users had reached 4.6 billion, which represents a global penetration rate of 58.4% and an increase of more than 200% over the last 10 years [2] (it should be noted that SNS users might not represent unique individuals and thus these data may not be a true reflection of the current number of global users). Not only are more people using SNSs, but users are also spending longer on these sites each day. Worldwide, the daily average time spent using SNSs has risen from 90 min in 2012 to 145 min in 2020 [3]. Of concern is that SNS use has been linked to reduced well-being and an increase in mental health disorders, particularly in younger populations [4,5,6], although such findings are the subject of debate [7,8]. Additionally, a recent meta-analysis suggests that between 5 and 25% of the global population report symptoms resulting from their SNS use that resemble criteria traditionally used to diagnose substance use disorders and other behavioural addictions [9]. Yet, other studies employing experimental methods offer a more sceptical view of the addictive potential of modern technologies [10,11,12,13], and these findings are mirrored by fears that we could be over-pathologising normal everyday activities [14,15,16].

Importantly, addictions are known to result in structural and functional adaptations in the brain that render the user more susceptible to the drug (or behaviour) and associated cues [17,18]. Neuroimaging studies have been instrumental in improving our understanding of the neural basis of substance use addictions [19] and have also provided key insights into the neural similarities and differences between the use of addictive substances and pathological behaviours (e.g., gambling disorder; [20]). For example, a study by Limbrick-Oldfield et al. [21] found that regions in the brain’s reward circuit (including the bilateral insula and ventral striatum) were activated in problem gamblers in response to cravings elicited by gambling-related images, which is similar to observations in substance use disorders [22,23]. Nonetheless, a recent meta-analysis comparing the activation likelihood estimation from fMRI studies of gambling disorder and alcohol use disorder during tasks of executive function revealed that the two conditions were associated with distinct changes in neural activity [24]. While gambling disorder was associated with activation in regions of the fronto-striatal reward network, alcohol use disorder was associated with both activations and deactivations of different nodes of this network. Additionally, research comparing brain structure between individuals with alcohol use disorder and problem gamblers has shown dissimilar brain morphology [25]. While alcohol use disorder was associated with reduced volume of brain regions involved in cognitive control and reward processing, no structural abnormalities were identified in problem gamblers. Such differences might reflect that behavioural addictions do not expose the brain to toxic chemical substances that may be responsible for the neuroadaptations associated with addictions to drugs. Neuroimaging techniques have thus provided a vital tool for improving our understanding of the neural correlates of different addictive disorders, indicating that similar as well as distinct neural abnormalities might underlie behavioural and substance-related addictions.

More recently, studies have begun to probe the neural underpinnings of SNS use behaviours. Based on neuroimaging research into offline social behaviours, the use of SNSs has been proposed to involve three key neural systems [26]: the *mentalising network* (i.e., dorsomedial prefrontal cortex, temporoparietal junction, anterior temporal lobe, inferior frontal gyrus, and the posterior cingulate cortex/precuneus) required for interpreting the emotions and mental states of others, the *self-referential cognition network* (i.e., medial prefrontal cortex and posterior cingulate cortex/precuneus) which enables self-reflections and social comparisons, and the *reward network* (i.e., ventromedial prefrontal cortex, ventral striatum, and ventral tegmental area) which is activated in response to social interactions. Given the pervasiveness of SNS use in society and the knowledge that a significant number of individuals report addiction-like symptoms, it seems also increasingly important to understand whether and how problematic SNS use might be reflected by structural and functional alterations in the brain. Recently we have argued for an incentive-sensitisation perspective to assessing the addictive potential of SNSs [27]. This theory of addiction posits that repeated drug use ‘hijacks’ the reward systems of the brain by sensitising them to the incentive properties of the drug, resulting in heightened drug ‘wanting’ without producing an increase in drug ‘liking’ [18,28]. In line with this, recent evidence has indicated that explicit cravings (i.e., ‘wanting’) are correlated with more problematic SNS use, independent of self-reported SNS liking [13,27]. Crucially, such an account would also predict adaptations of certain neural substrates in addicted SNS users. Thus, identifying neural similarities and differences between problematic SNS use and established addictive disorders would provide a valuable contribution in helping to determine whether some forms of SNS use might represent a behavioural addiction.

One way of investigating the neural correlates of SNS use is through the use of magnetic resonance imaging (MRI). Early neuroimaging studies have tended to investigate ‘internet addiction’ more broadly [29,30,31], and while the term ‘internet addiction’ is still commonly used to describe research investigating a range of problematic online activities, more recently, research has shifted away from this generalised construct to investigating specific online activities (e.g., online gaming, cybersex, social networking, online gambling, and online shopping; [32]). It therefore seems most appropriate to review the MRI literature for each of these behaviours in isolation when evaluating the addictive potential of a given activity. To that end, the present review will focus on discussing findings from studies that have specifically investigated SNS use behaviours, rather than internet or modern technology (e.g., smartphones) use more generally. Given that neuroimaging of ‘SNS addiction’ is still an emerging field, our review did not focus selectively on problematic (addictive) behaviours—as is typical in similar neuroscientific reviews of the drug addiction literature—but encompassed research that used MRI methods to study both problematic and nonproblematic (“healthy”) SNS use behaviours.

## 2. Method

### 2.1. Search Strategy

A literature search was conducted using the databases *Web of Science*, *PubMed*, and *Scopus.* The systematic review was conducted in accordance with the PRISMA statement (Preferred Reporting Items for Systematic Reviews and Meta-Analyses). The search was restricted to peer-reviewed research articles published in English between 2010 and 2022. The following search algorithm and terms were used: (“social media” OR “social network*” OR Facebook OR Twitter OR Instagram OR YouTube OR Snapchat OR TikTok) AND (MRI OR fMRI OR “magnetic resonance imaging” OR neuroimaging OR “BOLD signal” OR “BOLD response” OR “gray matter” OR “grey matter” OR “white matter”). The most recent search was conducted on the 25 October 2022 and returned 774 unique results. A systematic review protocol was not registered.

### 2.2. Study Selection

One author, MW, conducted the screening process and uncertainties regarding the inclusion of studies were resolved through consultation with NI. After removing duplicate studies, titles and abstracts were read and screened for relevance. Articles were excluded if they: (1) did not investigate the functional or anatomical brain correlates of SNS use using MRI methods; (2) only assessed online network size as an alternative index for real-life (offline) social networks and therefore did not directly investigate SNS use as a primary topic; (3) investigated social media marketing. Initial screening reduced the results to 39 potentially relevant articles. Upon closer inspection, a further 11 papers were removed after reading the full-length articles. These studies did not meet the above criteria as SNS use was not the primary topic of investigation or online social network size was used as a proxy for offline social networks. One study employing a ‘Tweet Task’ was considered for inclusion but deemed ineligible as the aim of the study focused on assessing how individuals from historically marginalised groups were affected by viewing discriminatory content shared by Donald Trump [33]. Both authors agreed that since the study did not report whether participants were SNS users themselves, and because neither regular nor problematic SNS use was assessed, the study was not suitable. Additionally, one study investigated virtual social rejection using a paradigm that simulated an online chatroom [34], but the authors agreed that this study did not specifically assess SNS use and thus was not included. Two studies (with the same sample) investigating problematic smartphone users [35,36] were included because participant recruitment focused on individuals who use smartphones for internet communication/social networking, and those who primarily used smartphones for other purposes (e.g., gaming) were excluded. Similarly, one study investigating the neural correlates of ‘mobile technology engagement’ [37] was included since the scale employed specifically assesses phone-based social media use and frequency of public status updating. Furthermore, a study investigating ‘specific internet addiction’ [38] was deemed eligible for inclusion since the sample comprised subgroups of individuals with internet gaming and social network addiction.

**Figure 1:**
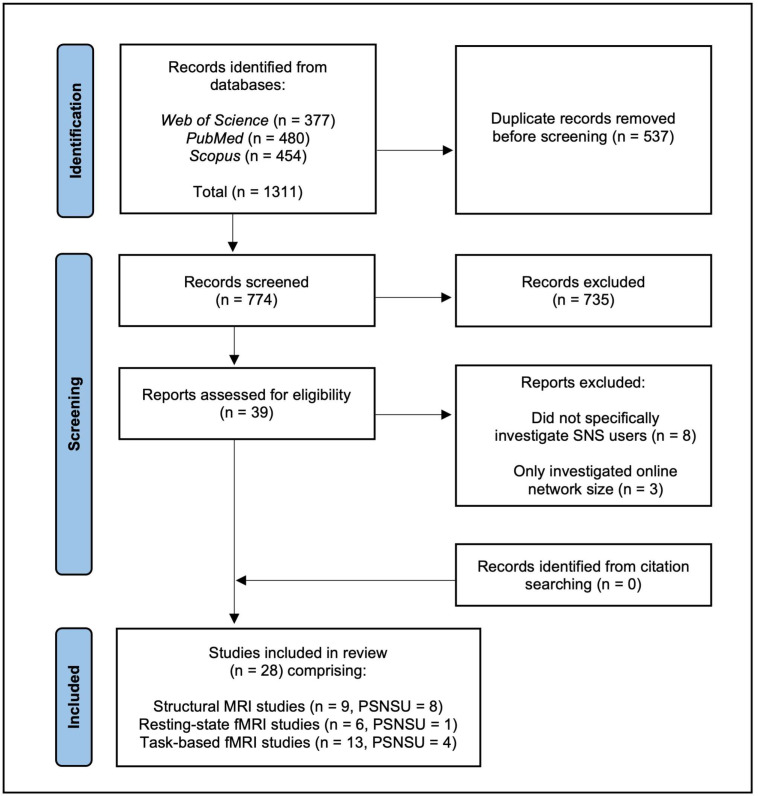
PRISMA flow diagram showing the process of the systematic literature search; PSNSU (problematic social networking site use) refers to the number of studies that investigated the neural correlates of problematic/compulsive SNS use.

The reference lists of included articles and previous systematic reviews of neuroimaging studies investigating internet addiction more broadly [39,40,41] were also hand-searched for any further relevant studies. However, no additional articles were deemed eligible for inclusion. Figure 1 illustrates our applied literature search using a PRISMA flow chart.

### 2.3. Data Extraction

Relevant data were extracted from each of the articles and summarised into tables according to the MRI method employed. The variables for which data were extracted included author names, year of publication, study design, number of participants, mean age, gender, type of SNS use, assessment tool for SNS use, MRI methodology and key findings (i.e., implicated brain regions). The data extraction process was conducted independently by MW.

### 2.4. Quality Assessment

Given that no standardised criteria exist for assessing the quality of neuroimaging studies, previous systematic reviews of MRI research have modified criteria from existing tools [42]. We opted to use a modified version of the Effective Public Health Practice Project (EPHPP) tool for quality assessment/risk of bias [43], similar to other systematic reviews of neuroimaging studies [44,45]. Our modified EPHPP comprised 10 quality criteria which assessed studies for selection bias, study design, neuroimaging methodology, and statistical analysis. Meeting a criterion was scored as 1 = ‘Yes’ or 0 = ‘No’ or ‘Unclear’, and ratings were summed to produce an overall quality score (range 0–10). A low (score = 0–4), moderate (score = 5–7) or high (score = 8–10) quality rating was then assigned to each study. The quality assessment was conducted independently by MW who consulted with NI to resolve any uncertainty.

## 3. Results

A total of 28 papers that met the eligibility criteria were identified and included in this review. The included studies were all published between 2013 and 2022 and comprised structural MRI studies (*n* = 9), resting-state fMRI studies (*n* = 6) and task-based fMRI studies (*n* = 13). Of the 28 studies only 13 investigated the neural correlates of compulsive or problematic SNS use (the majority of these being structural MRI studies, *n* = 8). All of the included studies were rated as moderate or high quality. Because of the heterogeneity of MRI methods, a meta-analysis was not conducted.

### 3.1. Structural MRI Studies

Of the nine structural MRI studies identified in the literature search, most employed a sample of Facebook users. The mean age of participants in the included studies ranged from 16 to 31.2. All but two studies used voxel-based morphometry (VBM) to assess grey matter volume (GMV), with one assessing changes in the surface area and cortical thickness of regions of interest (ROIs) using a longitudinal design and another study using diffusion tensor imaging (DTI) to assess the connectivity of white matter microstructure. Two studies were rated as moderate quality and seven studies as high quality. A summary of the included structural MRI studies is provided in Table 1.

Of the studies investigating GMV, the majority observed a negative correlation between excessive/problematic SNS use and GMV in regions that make up the brain’s reward systems. However, the implicated brain regions varied across studies and results are largely inconsistent. Three studies reported structural alterations of the nucleus accumbens (NAc; a component of the ventral striatum), which plays a key role in motivating reward-related behaviour. Montag et al. [46] found a negative correlation between bilateral NAc GMV and Facebook use intensity and reduced GMV in the right NAc was also associated with higher Facebook addiction scores. Correspondingly, He et al. [47] reported a negative correlation between GMV of the right ventral striatum and compulsive Facebook use. The only other study to report significant structural alterations of the NAc found that reduced GMV in this region was associated with higher use of the WeChat paying function [48]. However, in this instance, the specific association of smaller NAc GMV with the paying function and not with other functions (e.g., messaging and voice calling) or WeChat addiction scores could be argued to be more indicative of compulsive buying/shopping-related problems rather than as representing an SNS addiction. It has also been argued that the failure to observe reduced NAc structure for more problematic SNS users in other studies might indicate an important difference between SNS and substance use addictions [49]. Nonetheless, it is important to consider that the study by Montag et al. [46] employed a larger sample size (*n* = 62) and used the most objective index of Facebook use when compared to most other studies that have failed to observe this effect. In the study, participants installed a mobile app that tracked their use of Facebook across a 5-week period, whereas in all other structural MRI assays self-reports of usage/addiction severity were acquired. Thus, other studies may have been too underpowered to observe an effect or their reliance on self-report measures may have influenced the results.

Two studies found correlations between problematic SNS use and GMV of the anterior cingulate cortex (ACC) but in different directions. In their study of WeChat users, Montag et al. [48] found that reduced GMV of the subgenual ACC was associated with higher addiction scores and this relationship remained stable after controlling for age, gender, anxiety and depression. This finding seems to square with the important role of the ACC in the cognitive control network [50] and thus reduced ACC volume would likely result in the inhibitory control deficits exhibited by individuals with addictive disorders [51]. However, no other structural MRI study investigating SNS use has corroborated this finding and notably one study reports the opposite association. He, Turel and Bechara [49] found that increased GMV in the ACC and midcingulate cortex (MCC) was associated with more addictive Facebook use. The authors postulated that the finding could be the result of an adaptation and compensation process in which the ACC/MCC increases in volume to improve the efficiency of the inhibitory system in response to reduced GMV in other regions implicated in more problematic SNS use (e.g., amygdala). However, there are also important differences between these two conflicting studies that could potentially account for their results. Compared to Montag et al. [48], the study by He, Turel and Bechara [49] employed a substantially smaller sample size (61 vs. 20) and both assessed different SNS platforms, using different scales to assess addiction severity. Since Montag et al. [48] was the only study to investigate WeChat users, the findings could potentially highlight a difference between SNSs that are primarily used as a communication tool (i.e., for instant messaging) and other content sharing/microblogging based platforms. Additionally, Montag et al. [48] used a more specific ROI analysis to investigate subregional differences in ACC GMV. Findings were specific to the subgenual ACC and no correlations with other subregions of the ACC were found, whereas the positive correlation reported by He, Turel and Bechara [49] related to more dorsal ACC. Thus, the results might indicate important subregional specificity within the ACC, in which reduced subgenual ACC volume is related to more addictive SNS use.

Two studies by the same group reported reduced amygdala GMV in more excessive/problematic users [47,49]. The finding is therefore consistent with substance use addictions, in which addicts present reduced GMV of the amygdala [52]. However, unlike results typically observed in established addictions only one study observed reduced GMV in the prefrontal region, namely in the right orbitofrontal cortex (OFC) in problematic SNS users vs. healthy controls, matched for age, sex and IQ [35]. Furthermore, reduced GMV of the right OFC was the only significant difference between the two groups and results remained significant when controlling for potential comorbid conditions (i.e., depression, anxiety and alcohol addiction). While being the only structural MRI study to implicate this region in problematic SNS users the study did employ a comparatively large sample size (*n* = 88) and the results fit with findings from other addictive disorders that suggest an important role for the OFC in reward and decision-making processes.

In one of the few studies to find *positive* correlations between GMV and SNS use, Turel et al. [53] reported larger bilateral posterior superior temporal gyrus/middle temporal gyrus, and left posterior fusiform gyrus in more frequent Facebook users. It is suggested that increased GMV of these regions is associated with an improved ability to deal with social-semantic demands involved in Facebook use (e.g., recognising faces and interpreting the mental states of others). While no negative associations between GMV and SNS behaviours were observed, the study did not correlate GMV with a measure of SNS addiction, unlike most other structural MRI studies. Contrastingly, in another study by the same group but employing a measure of Facebook addiction, only negative correlations were observed [54]. In this study, participants completed a delay-discounting task before undergoing structural MRI. The results revealed a negative correlation between GMV in bilateral posterior insula (PI) and addiction scores, and this relationship was mediated by delay discounting, showing a stronger relationship in those participants with a preference for immediate rewards. It is suggested that the PI plays an important role in interoceptive awareness and thus promotes subjective feelings of urges and cravings in addiction [55]. Consequently, abnormal morphology of this region may result in deficits in decision making and inhibition, which is also corroborated by the negative correlation between PI GMV and delay discounting [54]. However, no other negative associations, such as those reported in other structural MRI studies and described above, were identified.

While the above studies are useful in demonstrating associations between SNS behaviours and brain morphology, they all share an important limitation in that they are unable to establish whether such structural abnormalities are directly caused by more frequent/addictive SNS use. Nonetheless, in the most recent structural MRI study to investigate SNS use, a longitudinal design was employed in which 189 adolescents were followed across three annual assessments [56]. The researchers used latent class growth curve analysis to assess how the development of cortical thickness and surface area of ROIs differed between high vs. low social media users. The results showed that the high social media use group had higher baseline cortical thickness in the lateral and medial prefrontal cortex (L/MPFC). Over three years, the high social media use group also showed a stronger reduction in the cortical thickness of the lateral prefrontal cortex and reduced surface area of the temporal parietal junction (TPJ). While such findings are consistent with the researchers’ hypothesis that more excessive SNS use should result in accelerated thinning of regions involved in processing social information (e.g., TPJ) and cognitive control (e.g., LPFC), these associations did not remain significant after correcting for false discovery rate (FDR). Thus, the findings only provide limited evidence that more excessive SNS use might be directly responsible for developmental changes in brain structure.

In the only study using DTI to assess white matter connectivity, He et al. [57] found increased mean diffusivity (MD) in the body and splenium of the corpus callosum in more excessive social media users. While this finding is consistent with those reported in other addictive disorders, the authors note that the results did not reach significance when a more conservative whole-brain voxel-wise analysis (using Tract Based Spatial Statistics; TBSS) was applied. However, the whole-brain analysis did reveal that more excessive SNS use was associated with increased MD in the forceps minor and ventral semantic path. The forceps minor is a white matter bundle that connects the two frontal lobes and thus increased MD in this region is indicative of inter-hemispheric communication deficits between the frontal lobes. Given the important role the frontal lobes play in cognitive control and reward processing, it seems plausible that more problematic SNS users might exhibit less efficient connectivity in these regions. However, since the study by He, Turel and Bechara [57] is so far the only study investigating white matter microstructure in SNS users, and also given the relatively small sample size employed (*n* = 20), more research is required to validate these findings. Furthermore, TBSS is not without limitations as there is potential for bias in anatomical specificity and accuracy during the skeleton projection step. Future studies should consider using more reliable methods to achieve a more comprehensive understanding of potential alterations in white matter connectivity underlying problematic SNS use.

**Table 1:** Summary of structural MRI studies.

Authors (Year)	Sample	Mean Age	PSNSU	SNS Assessment Tool	Design	Main Results	Quality Assessment
Achterberg et al. (2022) [56]	189 individuals categorised into high (*n* = 52, females = 68%) vs. low (*n* = 137, females = 45%) SNS use groups.	10–25	Yes	Modified Compulsive Internet Use Scale [58].	Longitudinal design (3 annual scans). Assessed cortical thickness and surface area of ROIs.	The high (vs. low) group had thicker lateral and medial PFC at baseline. The high group showed a faster reduction in LPFC and TPJ over 3 years (non-FDR corrected).	High
He, Turel and Bechara (2017) [49] ^a^	20 Facebook users (females = 10).	20.3	Yes	Modified Compulsive Internet Use Scale [58].	GMV was used to predict the severity of SNS addiction.	SNS addiction was associated with reduced GMV in the amygdala but increased GMV in the ACC/MCC.	Moderate
He et al. (2017) [47]	50 Facebook users categorised into excessive (*n* = 25) vs. non-excessive (*n* = 25) use groups, with 8 females in each group.	27	Yes	Modified Compulsive Internet Use Scale [58].	GMV was correlated with excessive SNS use scores and differences between groups were compared.	Excessive use group had reduced GMV in the bilateral amygdala and right ventral striatum. GMV of these regions were negatively correlated with excessive use scores.	High
He et al. (2018) [57] ^a^	Sample same as He, Turel and Bechara [49].	20.3	Yes	Modified Compulsive Internet Use Scale [58].	DTI was used and ROI analysis was performed using subregions of the corpus callosum. Supplementary whole-brain voxel-wise analysis (using Tract Based Spatial Statistics; TBSS) also performed.	Excessive SNS use associated with reduced white matter connectivity in the body and splenium of the corpus callosum (non-FDR corrected). TBSS analysis revealed reduced connectivity in the forceps minor and ventral semantic pathway.	Moderate
Lee et al. (2019) [35] ^b^	88 smartphone users categorised as healthy controls (*n* = 49, females = 17) vs. problematic users (*n* = 39, females = 10).	22.6	Yes	Korean Smartphone Addiction Proneness Scale [59].	ROI analysis performed on fronto-cingulate region. Differences in GMV and correlations with addiction severity analysed. Subsequent whole-brain analysis performed.	ROI analysis revealed smaller right OFC GMV in problematic users. Reduced GMV in the OFC correlated with higher addiction scores.	High
Montag et al. (2017) [46]	62 Facebook users (females = 25).	23.2	Yes	Online Social Network Addiction Scale [60].	Mobile app tracked participants Facebook usage across 5-weeks. Facebook use and SNS addiction were correlated with GMV in the NAc.	Left and right NAc GMV was negatively correlated with Facebook use. Reduced GMV in the right NAc was associated with more addicted Facebook use.	High
Montag et al. (2018) [48]	61 WeChat users (females = 21).	22.3	Yes	Modified short Young’s Internet Addiction Test [61].	WeChat use intensity and addiction severity were correlated with GMV.	WeChat addiction was negatively correlated with subgenual ACC GMV. Reduced GMV in the NAc was associated with higher usage of WeChat’s paying function (but not WeChat addiction).	High
Turel et al. (2018a) [53]	33 Facebook users (females = 21).	23.1	No	N/A	Using a whole-brain analysis, correlations between GMV and Facebook use were assessed whilst controlling for age and sex.	Three clusters of GMV: bilateral posterior superior temporal gyrus/middle temporal gyrus (pSTG/MTG), and left posterior fusiform gyrus, were positively correlated with Facebook use.	High
Turel et al. (2018b) [54]	32 Facebook users (females = 6).	31.2	Yes	Modified Online Video Game Addiction Scale [62].	Computer-based delay discounting task completed before MRI. Using a whole-brain and ROI analysis, GMV was correlated with SNS addiction and delayed discounting whilst controlling for age and sex.	ROI analysis revealed negative correlations between GMV in left and right posterior insula (PI) and delayed discounting as well as addiction. Delayed discounting mediated the relationship between SNS addiction and reduced GMV in left/right PI.	High

*Note.* PSNSU refers to studies that investigated the neural correlates of problematic/compulsive SNS use. ^a^ He, Turel and Bechara [49] and He et al. [57] used the same sample of Facebook users (*n* = 20) as a task-based fMRI study by Turel et al. [63]. ^b^ Lee et al. [35] used the same sample of smartphone users (*n* = 88) as a resting-state fMRI study by Lee et al. [36]. Although SNS use was not assessed directly, participants who used smartphones primarily for other purposes (e.g., gaming) were excluded.

### 3.2. Resting-State fMRI Studies

Six studies were identified that used resting-state fMRI data to investigate the neural correlates of SNS use. Two studies (with the same sample) recruited high vs. low SNS users based on time spent using three popular Chinese SNSs (Weibo, TikTok and Kwai), one study employed a sample with problematic SNS users, one study employed a sample of Facebook users, another study employed a sample of participants with varying levels of mobile technology engagement and a final study employed a sample of Weibo users. The mean age of participants ranged from 20.9 to 25.7. Three studies were rated as moderate quality and three as high quality. A summary of the included resting-state fMRI studies is provided in Table 2.

In the only study to measure resting-state fMRI data in a sample of problematic SNS users, Lee et al. [36] employed functional connectivity analysis at seeds in the dorsal attention network (DAN) and the ventral attention network (VAN). No significant differences between problematic SNS users and healthy controls emerged when using functional connectivity analysis with VAN seeds. However, when using seeds in the DAN, problematic SNS users were shown to have stronger functional connectivity between the right intraparietal sulcus and the right middle occipital gyrus. Because the middle occipital gyrus plays an important role in sensory processing the authors suggested that abnormal connectivity between this region and the DAN may interfere with attentional control processes in problematic SNS users. In addition, when compared to controls functional connectivity between the right frontal eye field and the right dorsolateral prefrontal cortex (DLPFC) was weaker in problematic SNS users. Given the critical role of the DLPFC in exerting executive control, a less efficient control network may consequently result in the inability to manage the amount of time spent on SNSs resulting in more problematic use.

In a study employing a sample of Facebook users, Meshi et al. [64] investigated whether resting-state fMRI data correlated with the frequency users shared personal information on Facebook. Participants completed a self-related sharing assessment [65] and scores were correlated with functional connectivity using seeds at the medial prefrontal cortex (MPFC), central precuneus (CP), caudal anterior cingulate cortex (cACC), and the ventral striatum. No significant correlations were observed when using seeds at the cACC and ventral striatum. However, intrinsic functional connectivity between the MPFC and the right DLPFC, as well as between the CP and right DLPFC, were both positively correlated with self-related sharing. Additionally, connectivity between the CP and the left lateral OFC was positively correlated with self-related sharing, although connectivity between the CP and the left anterior temporal pole (ATP) was negatively correlated with self-related sharing. Increased functional connectivity of both the MPFC and CP to other brain regions is in line with the suggested view that these regions play a role in self-referential processing [66]. In particular, since the DLPFC is critical for executive functioning and working memory, stronger functional connectivity to this region might indicate a greater ability to hold self-related information in working memory, in turn facilitating self-related sharing.

A study by Wilmer et al. [37] investigated functional connectivity in two dissociable pathways stemming from the ventral striatum. While higher connectivity between the ventral striatum and the ventromedial prefrontal cortex (VMPFC) was associated with higher mobile technology engagement (including increased social media use and more frequent status updates), connectivity between the ventral striatum and DLPFC was associated with reduced mobile technology engagement. The researchers suggest that these findings corroborate the notion of the ventral striatum-VMPFC pathway being involved in reward processing, thus resulting in greater sensitivity to rewards obtain through mobile technologies. Similarly, the association between increased functional connectivity in the ventral striatum-DLPFC pathway and reduced mobile media engagement is consistent with this networks role in exerting executive control over behaviour, resulting in more controlled use of mobile technologies.

Using seeds at the medial prefrontal cortex (MPFC), dorsomedial prefrontal cortex (DMPFC) and temporoparietal junction (TPJ), Zhang and Mo [67] correlated functional connectivity with participant’s reposting rate in an experimental paradigm that simulated Weibo use. During the task, completed after the scanning session, participants were shown a series of 90 Weibo messages of either positive, negative or neutral valence. After reading each Weibo message participants had to decide whether to ‘repost’ or ‘not repost’ the message. It was shown that overall participants preferred to repost negative, compared to positive or neutral messages. The repost rate of negative messages was positively correlated with functional connectivity between left TPJ and left middle frontal lobe, as well as between the left TPJ and right insula. Additionally, functional connectivity between left DMPFC and medial OFC was also shown to be positively correlated with the repost rate of negative messages. Conversely, the repost rate of positive messages was positively correlated with functional connectivity between the right TPJ and right superior temporal lobe. Finally, the repost rate of neutral messages showed a positive correlation with the functional connectivity between left TPJ and left ventrolateral OFC, in addition to the functional connectivity between bilateral DMPFC. The finding of increased functional connectivity between the TPJ and other regions being associated with an increased repost rate across all message types was argued to corroborate the important role that the TPJ plays in social communication.

A recent study by Hu et al. [68] used dynamic functional network connectivity analysis to investigate the brain dynamics of reading SNS posts on a smartphone. Resting-state fMRI was first recorded at baseline before participants were assigned to either a social media or science fiction reading task, which took place outside of the scanner. Immediately after the reading task participants underwent a second fMRI session. Reading social media posts was shown to decrease functional connectivity between the default mode network (DMN) and frontoparietal network (FPN), but increased connectivity between the DMN and visual network. It is suggested that since a main function of the DMN is mind wandering [69], SNSs might stimulate the visual network to induce mind wandering, resulting in increased inattention after SNS use. In another study by the same group and recruiting from the same pool of participants, Hu et al. [70] used a longitudinal design to investigate changes in functional connectivity after a month of excessive SNS use. The results showed that the functional connectivity of light SNS users was more similar to that of heavy users after they completed the four-week period of increased SNS use. Using inter-subject correlation analysis, increased SNS use was shown to have a widespread impact on almost all brain networks and functional connectivity between regions that were most affected were associated with selective attention. The study is the first to indicate that more excessive SNS use can result in alterations of cerebral functional connectivity when assessed longitudinally, and results therefore warn of potential neurobiological consequences attributable to just four weeks of excessive SNS use.

**Table 2:** Summary of resting-state fMRI studies.

Authors (Year)	Sample	Mean Age	PSNSU	SNS Assessment Tool	Design	Main Results	Quality Assessment
Hu, Cui et al. (2022) [68]	70 males categorised as heavy (*n* = 30) vs. light (N = 40) SNS users based on time spent on Weibo, TikTok, and Kwai.	20.9	No	N/A	Participants surfed Weibo or read a science fiction novel on their phone after baseline recording and before a second fMRI recording.	Reading SNS posts reduced functional connectivity between the default mode network (DMN) and frontoparietal network (FPN), but increased connectivity between the DMN and visual network.	Moderate
Hu, Yu et al. (2022) [70]	49 males recruited from the same pool as Hu, Cui et al. [68], categorised as heavy (N = 30) vs. light (N = 19) SNS users.	21	No	N/A	Longitudinal design. Light SNS users were instructed to increase their use to 2 h per day for 4 weeks before undergoing a second fMRI session.	Difference in functional connectivity between two groups was attenuated after light users increased their SNS use. Increased SNS use had widespread impact on almost all brain networks.	High
Lee et al. (2021) [36] ^a^	88 smartphone users categorised as healthy controls (*n* = 49, females = 17) vs. problematic users (*n* = 39, females = 10).	22.6	Yes	Korean Smartphone Addiction Proneness Scale [59].	Analysed ROIs in the dorsal and ventral attention network.	Problematic users had increased functional connectivity between the right middle occipital gyrus and the right intraparietal sulcus but reduced functional connectivity between the right frontal eye field and right dorsolateral prefrontal cortex.	High
Meshi et al. (2016) [64]	35 Facebook users (females = 21).	25.7	No	Self-Related Sharing Assessment [65].	fMRI data was correlated with the degree to which participants share self-related information on Facebook.	Functional connectivity between the MPFC and right DLPFC, CP and right DLPFC as well as between the CP and left OFC was associated with sharing personal information on Facebook. Whereas connectivity between the CP and left ATP was negatively associated with self-related sharing score.	High
Wilmer et al. (2019) [37]	26 healthy SNS users (females = 15).	21.4	No	Mobile Technology Engagement Scale [71].	Functional connectivity was assessed at ventral striatum, ventral medial PFC and DLPFC.	Higher connectivity between the ventral striatum and the ventral medial PFC was associated with greater mobile technology engagement. However, higher connectivity between the ventral striatum and DLPFC was associated with lower engagement.	Moderate
Zhang and Mo (2016) [67] ^b^	28 Weibo users (females = 14).	21.2	No	N/A	Functional connectivity was assessed at MPFC, DMPFC and TPJ. After fMRI participants completed an experimental task in which they read valenced Weibo messages and decided whether to ‘repost’ or ‘not repost’ each message.	Reposting positive messages was associated with increased connectivity between right TPJ and right superior temporal lobe. Greater connectivity between left TPJ, left middle frontal lobe and right insula, and between left DMPFC and medial OFC were positively correlated with reposting negative messages. Connectivity between left TPJ and left ventrolateral OFC, and between bilateral DMPFC was positively correlated with reposting neutral messages.	Moderate

*Note.* PSNSU refers to studies that investigated the neural correlates of problematic/compulsive SNS use. ^a^ Lee et al. [36] used the same sample of smartphone users (*n* = 88) as a structural MRI study by Lee et al. [35]. Although SNS use was not assessed directly, participants who used smartphones primarily for other purposes (e.g., gaming) were excluded. ^b^ Zhang and Mo [67] used the same sample of Weibo users (*n* = 28) as a task-based fMRI study by Zhang and Qu [72].

### 3.3. Task-Based fMRI Studies

The 13 task-based fMRI studies identified in the literature search used a range of different experimental tasks, thus making it difficult to directly compare neural activity between studies. Two studies used cognitive control tasks (Emotional Stroop and Go/No-Go), two studies used a self-retrieval/self-concept paradigm, another used a description task, and the remaining eight studies all used variations of a cue reactivity task or a paradigm that simulated SNS use. The specific SNS platform under investigation also varied between studies. Four studies employed a sample with problematic SNS users, with the remaining studies investigating either Facebook, Instagram, TikTok, Weibo or general SNS users. The mean age of participants ranged from 16.8 to 26.1. Four studies were rated as moderate quality and nine as high quality. A summary of the included task-based fMRI studies is provided in Table 3.

Dieter et al. [38] investigated neural differences in emotional inhibitory control processing between individuals with a gaming addiction (*n* = 13) or social network addiction (*n* = 12) and healthy controls (*n* = 23). During fMRI the participants completed an Emotional Stroop Task with four categories of positive, negative, neutral and socially anxious words. Behavioural results revealed no significant difference in the reaction times to emotional words between healthy controls and internet addicts (gaming and social network addicts). Similarly, and in contrast to what was hypothesised, the fMRI data revealed no significant difference in dorsal ACC activation between controls and specific internet addicts during socially anxious word blocks. Nonetheless, it was shown that compared to individuals with a social network addiction, participants with an internet gaming addiction exhibited reduced activation in left middle and superior temporal gyrus during socially anxious words. However, all other between group comparisons of the subgroups revealed no significant differences in activation. The findings were interpreted as social anxiety-related alterations in response inhibition playing a larger role in internet gaming addiction rather than SNS addiction, whereby gaming might represent a coping strategy to avoid face to face interactions. The only other study to employ an established reaction time task investigated inhibitory control using a Facebook-specific Go/No-Go paradigm. In this study, Turel et al. [63] observed that bilateral ventral striatum activity during Facebook-Go trials was positively correlated with addiction scores. However, contrary to the hypothesis, activity in regions that make up the brain’s inhibitory system (prefrontal cortex) during Facebook-No-Go trials were not negatively correlated with addiction severity. This pattern of results suggests that on the one hand, similar to other addictive disorders, Facebook users with more addiction-like symptoms exhibit a hyperactive amygdala-striatal (impulsive) brain system, whereas on the other hand they do not have a hypoactive prefrontal inhibitory system, which is dissimilar to other addictions.

Alterations of ventral striatal activity have also been reported in other fMRI studies investigating SNS use. One study employing a sample of Facebook users recorded neural activity whilst participants completed a ‘description task’ [73]. Here, participants received a description (e.g., intelligent) that appeared with a picture of themselves or another person, with the descriptions ostensibly provided by another participant. The findings revealed that when receiving gains in reputation (vs. observing the gains in reputation of another) activity in the left NAc (a component of the ventral striatum) predicted more intense Facebook use. Furthermore, Meshi, Morawetz and Heekeren [73] found that left NAc activity in response to monetary rewards did not predict more intense Facebook use, indicating that individual sensitivity of the NAc in processing social information relevant to the self (rather than its sensitivity to reward more generally) influences the use of Facebook.

Sherman and colleagues also observed alterations in NAc activity in their studies which analysed data from the same sample of teenaged Instagram users. In their experiment, participants completed a paradigm that simulated Instagram use, in which they viewed a series of photos (including some of their own photos) with varying numbers of ‘likes’. Each image was ostensibly rated by other participants and appeared with either a high or low like count. After viewing each image participants had to decide themselves whether to like the picture or not. In their first paper which analysed data from 32 teenagers (mean age = 16.8), Sherman et al. [74] reported increased bilateral NAc activation when participants viewed their own photos that had received many (compared to few) likes. Additionally, when participants viewed ‘risky’ photos (e.g., images depicting cannabis use) they exhibited hypoactivity in regions implicated in cognitive control (e.g., dorsal ACC, bilateral PFC, and lateral parietal cortex). In their later paper, the authors included data from an additional 26 university students (mean age = 19.9) who underwent the same procedure [75]. The findings revealed that university students displayed a similar pattern of results, showing increased activity in bilateral NAc when viewing their own photos with many (vs. few) likes. While this effect replicated the previous study using an older sample, unlike the teenaged Instagram users, the university students did not display reduced activity in regions involved in cognitive control whilst viewing risky images. In the last paper published by this group, Sherman et al. [76] analysed neural activity (in the same sample of Instagram users) when participants decided to like another’s photo. Consistent with their hypothesis liking an image was associated with increased activity in the ventral striatum and VMPFC, as well as a range of other structures involved in reward, salience processing and executive function. The NAc/ventral striatum therefore appears to be a key region involved in facilitating SNS use by becoming hyperactive when receiving peer feedback (e.g., obtaining likes), providing peer evaluation (e.g., distributing likes) and responding to SNS cues (e.g., during SNS-Go trials in the Go/No-Go task).

In a similar experiment to that of Sherman and colleagues, Nasser et al. [77] employed a sample of problematic (*n* = 15) and non-problematic (*n* = 15) Instagram users who completed a paradigm that mimicked Instagram use whilst undergoing fMRI. During the task participants viewed a series of Instagram photos that were categorised as either risky (e.g., a selfie whilst driving), neutral (e.g., greyscale image of an inanimate object), or positive (i.e., photos from the participants own Instagram account), and each appeared with either a high or low number of ‘likes’. After viewing each image participants also had to decide whether to ‘like’ or ‘pass’ the photo. Behaviourally it was found that problematic Instagram users liked significantly more risky pictures than the control group. Analysis of the fMRI data also revealed that compared to non-problematic Instagram users, problematic users exhibited increased bilateral precuneus activation when viewing risky images. Increased precuneus activity in more problematic users is assumed to reflect the role this region plays in assigning salience and responding to habit-forming stimuli [78], which is corroborated by the behavioural results (as problematic users were more responsive to risky images). Additionally, activity in the right MPFC when viewing risky photos was negatively correlated with Instagram addiction scores, suggesting that more problematic users might experience decision making/cognitive control deficits when viewing addiction-related cues.

As well as investigating peer feedback in the form of SNS ‘likes’, other studies have investigated neural reactivity to receiving comments on SNS posts. In a task simulating the use of Facebook, adolescent SNS users had to post controversial statements (e.g., “abortions should be illegal”) to a Facebook group and received either positive or negative comments on their post from peers [79]. When compared to a control condition (i.e., posting neutral statements and receiving neutral feedback) trials containing emotionally valenced statements elicited activation in the MPFC, precuneus and PCC. Additionally, receiving negative (vs. positive) peer feedback was associated with increased activity in the ventrolateral PFC, MPFC, and anterior insula, whereas viewing positive comments was associated with greater activity in regions including the posterior insula, TPJ, precuneus and PCC. However, the amount participants used SNSs in the real world did not interact with these effects.

Similarly, fMRI cue-reactivity paradigms have also been utilised to investigate the neural regions involved in decisions to repost content on microblogging platforms (e.g., Weibo), where short passages of text are typically shared by users. Analysing data from the same sample as a previous resting-state fMRI study [67], Zhang and Qu [72] investigated task-based neural activity whilst participants completed a paradigm simulating Weibo use. When reposting emotionally valenced (vs. neutral) messages participants showed increased activity in regions implicated in the emotion and cognitive control systems (e.g., DLPFC, insula, precuneus and TPJ), suggesting that these messages recruit more cognitive and emotional resources. Behaviourally participants were more likely to repost negatively valenced messages, which was also reflected by an increase in TPJ activation. Since the TPJ is known to play a key role in social communication and mentalising, these findings also imply that this region is involved in decisions to promulgate negative information online.

In another fMRI study using an SNS exposure paradigm, 30 TikTok users were shown six personalised recommended TikTok videos (based on user-specific preferences) that were extracted from their own account as well as six generalised recommended videos for new users [80]. Watching personalised compared to generalised recommended TikTok videos resulted in increased activation in regions of the default mode network (DMN; including the bilateral superior and middle temporal gyri, temporal pole, ventral PCC, MPFC, and angular gyrus) as well as in the left dorsal lateral and inferior frontal regions, anterior thalamus and cerebellum. In addition, voxel-wise psychophysiological interaction analysis was used to assess task-related connectivity changes between seeds in the DMN and other regions. The analysis revealed increased connectivity between the DMN seeds and regions including the visual network, primary auditory cortex, and middle frontal gyrus, but reduced connectivity with the cingulate cortex, cuneus and inferior parietal lobe when watching personalised (vs. generalised) videos. Finally, activation in regions implicated in addiction and reward learning (ventral tegmental area, substantia nigra and NAc) were also explored using ROI analyses. While the substantia nigra was activated by both personalised and generalised videos, increased ventral tegmental activation was specific to watching personalised videos. Interestingly, however, and contrasting with an important role for the NAc in other SNS use behaviours, the NAc was deactivated (although not significantly) when viewing both video types. In an extension of this work, the same group published further analyses of the same dataset using graph theory to investigate the functional connectivity between seven networks [81]. Results revealed that viewing personalised videos increased connectivity in the DAN-VAN-DMN pathway, whereas both video types resulted in reduced coupling between the salience network (i.e., anterior insula and dorsal ACC) and VAN as well as between two subsystems in the DMN. Taken together, these findings support a role of the DMN in self-relevant information processing, whereby regions in the DMN are activated by user-specific content and may also facilitate prolonged SNS use through increased coupling of the DMN to visual and auditory pathways, but reduced coupling to regions in the control network. The authors suggest that the results are also consistent with roles for the substantia nigra in saliency-coding and ventral tegmental area in reward-value coding.

In a study by Leménager et al. [82], participants completed a self-retrieval paradigm in which they rated the extent to which various self-concept-related characteristics described their self, ideal self, and gaming avatar (created by them), whilst undergoing fMRI. The sample consisted of pathological internet gamers (*n* = 19), pathological SNS users (*n* = 19) and healthy controls (*n* = 19). It was found that individuals with a gaming addiction exhibited increased left angular gyrus activation when reflecting on the characteristics of their avatar, whereas individuals with an SNS addiction exhibited striatal hypoactivations whilst making self (vs. ideal)-reflections. The authors suggest that the finding of reduced activity in the dorsal striatum in participants with an SNS addiction compared to healthy controls might indicate that self (vs. ideal)-reflections are less rewarding for SNS addicts. This in turn might suggest that these individuals experience deficits in emotion regulation that could be the result of (or the perception of) social feedback received online. A more recent study used a similar self-concept paradigm in which participants had to make self-judgements on academic, physical and prosocial traits from their own perspective and from the perspective of others [83]. It was shown that individuals who reported less SNS use made more positive ratings from self-judgements vs. reflected-peer-judgements and more excessive SNS use was linked to increased MPFC activity during self-judgements and particularly during physical (vs. academic and prosocial) self-judgements. Nonetheless, longitudinal assessments of clinical symptoms, prosocial behaviour and self-concept clarity (at 1-and 2-year follow up) indicated no long-term effects of SNS use or altered MPFC activity. Thus, while the findings are consistent with the MPFC’s core role in the self-referential cognition network and suggest that excessive SNS use might modulate activity in this region during self-judgements resulting in more intensified self-refection processes, there is no evidence that this leads to negative long-term consequences.

**Table 3:** Summary of task-based fMRI studies.

Authors (Year)	Sample	Mean Age	PSNSU	SNS Assessment Tool	Design	Main Results	Quality Assessment
Dieter et al. (2017) [38]	48 participants comprising healthy controls (*n* = 23, females =13), internet gaming addicts (*n* = 13, females = 2) and SNS addicts (*n* = 12, females = 6).	25.9	Yes	Assessment of Internet and Computer game Addiction checklist [84].	Participants completed an Emotional Stroop Task (EST) with socially anxious, positive, negative and neutral words whilst undergoing fMRI.	No group differences in ACC activity were found. When viewing socially anxious words internet gaming addicts had reduced activity in the left middle and superior temporal gyrus compared to social network addicts.	High
Leménager et al. (2016) [82]	57 participants comprising healthy controls (*n* = 19, females = 12), pathological internet gamers (*n* = 19, females = 5) and pathological SNS users (*n* = 19, females = 10).	26.1	Yes	Assessment of Internet and Computer game Addiction checklist [84].	While undergoing fMRI participants completed a self-retrieval paradigm, in which they rated the extent to which self-concept-related characteristics described their self, ideal, and gaming avatar.	When making self-reflections (vs. ideal-reflections) pathological SNS users had reduced activation in the dorsal striatum, thalamus and the inferior/middle frontal gyrus (vs. controls). Reduced activity in these regions was correlated with symptom severity.	Moderate
Meshi, Morawetz and Heekeren (2013) [73]	31 Facebook users (females = 17).	23.1	No	Facebook Intensity Scale [85].	While undergoing fMRI participants completed a task in which they received gains in reputation and observed the gains in reputation of another person, and a separate card task where they played for a monetary reward.	Activity in the left NAc in response to gains in self reputation (vs. observing reputation gains of others) predicted Facebook use. Whereas activation of the NAc in response to monetary gains did not predict Facebook use.	High
Nasser et al. (2020) [77]	30 Instagram users categorised as problematic users (*n* = 15, females = 5) vs. healthy controls (*n* = 15, females = 8).	21.9	Yes	Modified short Young’s Internet Addiction Test [61].	Participants completed a fMRI-based cue reactivity task with negative or positive valenced Instagram selfie images and neutral landscape image cues.	Negatively valenced cues produced increased activation of the precuneus in problematic users. Activation in the right MPFC in response to addiction-related cues was negatively correlated with Instagram addiction score.	Moderate
Peters et al. (2021) [83]	150 healthy adolescents and young adults (11–21 years old, females = 80).	15.7	No	N/A	During fMRI, participants rated themselves on 60 traits related to academic, physical and prosocial characteristics, and also indicated how their peers would judge them on the same traits. Longitudinal questionnaires assessing positive and negative outcomes were collected at 1- and 2-year follow-up.	Increased SNS use was associated with medial PFC activity during self-judgements (vs. reflected-peer-judgements), and increased activity when making judgements about physical traits. SNS use or medial PFC activity had no long-term effect on clinical symptoms, prosocial behaviour or self-concept clarity.	High
Sherman et al. (2016) [74] ^a^	32 Instagram users (females = 18).	16.8	No	N/A	Participants completed a paradigm that simulated Instagram use whilst undergoing fMRI. ‘Instagram photos’ were displayed (including some of the participants own photos), and each appeared with a high or low number of likes. Participants decided whether to like each photo or move on.	Viewing photos with a high number of likes was associated with increased activity in the precuneus, MPFC, left temporal pole, lateral occipital cortex, hippocampus, NAc, caudate, putamen and thalamus. Participants had greater activity in bilateral NAc when viewing own photos with many likes. Decreased activity in regions implicated in cognitive control was found when participants viewed risky photos.	High
Sherman, Greenfield et al. (2018) [75] ^a^	58 Instagram users, comprising 32 high school (females = 18) and 26 university students (females = 17).	16.8 vs. 19.9	No	N/A	The fMRI task was the same as Sherman et al. [74].	Replicating Sherman et al. [74], university students showed greater bilateral NAc activity when viewing their own photos that had received many likes. Bilateral NAc activity increased linearly with age in the high school sample, but not in the university sample. Viewing risky images was not associated with decreased activity in regions responsible for cognitive control in the university sample.	High
Sherman, Hernandez et al. (2018) [76] ^a^	Same sample as Sherman, Greenfield et al. [75].	16.8 vs. 19.9	No	N/A	The fMRI task was the same as Sherman et al. [74]; however, in this study, the researchers examined neural responses when participants provided ‘likes’ to others, rather than when they viewed the images.	When participants ‘liked’ a photo they showed activation in the ventral striatum, vmPFC, dorsal striatum, thalamus, bilateral insula/OFC, hippocampus, amygdala, ACC, inferior frontal gyrus and the bilateral intraparietal sulcus.	High
Su, Zhou, Gong et al. (2021) [80] ^b^	30 TikTok users (females = 14).	23.7	No	Modified Young’s Internet Addiction Test [86].	Whilst undergoing fMRI participants were shown TikTok videos including generalised recommended videos for new users and personalised recommended videos for experienced users (taken from their personal TikTok account). Participants had not seen any of the videos before entering the scanner.	When viewing personalised videos participants showed increased activity in parts of the DMN. Parts of the DMN also showed enhanced coupling with primary visual and auditory areas, and decreased coupling with precuneus and cingulate cortex when viewing personalised videos.	High
Su, Zhou, Wang et al. (2021) [81] ^b^	Same sample as Su et al. [80].	23.7	No	Modified Young’s Internet Addiction Test [86].	The fMRI task was the same as Su et al. [80]. However, in this study, the researchers used graph theory analysis to investigate functional connectivity between ROIs within the DMN, dorsal and ventral attentional networks (DAN/VAN), the frontal-parietal network (FPN) and the salience network (SN).	Both personalised and generalised videos enhanced connectivity in the DAN-FPN-DMN pathway. Whereas personalised videos also increased connectivity in the DAN-VAN-DMN pathway. Additionally, both video types resulted in reduced coupling between the SN and VAN as well as between two subsystems in the DMN.	High
Turel et al. (2014) [63] ^c^	20 Facebook users (females = 10).	20.3	Yes	Modified Online Video Game Addiction Scale [62].	Participants completed a Facebook-specific Go/No-Go task whilst undergoing fMRI.	Bilateral ventral striatum activity on Facebook-go trials was positively correlated with addiction scores. However, activity in the inhibitory control system (i.e., ACC and PFC) on Facebook-No-Go trials was not correlated with addiction score.	Moderate
Wikman et al. (2022) [79]	92 healthy adolescents and young adults (17–20 years old, females = 52).	18.7	No	N/A	Participants completed a task simulating Facebook use whilst undergoing fMRI. Participants had to agree or disagree with either neutral or controversial statements and then received positive or negative peer feedback on their opinions in the form of Facebook comments.	Negative feedback was associated with increased VLPFC, MPFC, and anterior insula activity. Positive feedback was associated with greater activity in regions including the posterior insula, TPJ, precuneus and PCC. No association with real-world SNS use.	High
Zhang and Qu (2020) [72] ^d^	28 Weibo users (females = 14).	21.2	No	N/A	Participants viewed positive, negative and neutral microblog messages mimicking those on Weibo whilst undergoing fMRI. Participants decided whether to repost or not repost each microblog.	Reposting negative microblogs increased activity in postcentral gyrus, superior frontal gyrus and TPJ. Reposting emotionally valenced (vs. neutral) microblogs resulted in increased activity in DLPFC, insula, precuneus and TPJ.	Moderate

*Note.* PSNSU refers to studies that investigated the neural correlates of problematic/compulsive SNS use. ^a^ Sherman et al. [74,75,76] used the same sample of high school students (*n* = 32) and Sherman et al. [75,76] used the same sample of university students (*n* = 26). ^b^ Su et al. [79,80] used the same sample of TikTok users (*n* = 30). While this study administered an assessment of ‘TikTok addiction’, scores were not correlated with brain activity. ^c^ Turel et al. [63] used the same sample of Facebook users (*n* = 20) as two structural MRI studies by He, Turel and Bechara [49] and He et al. [57]. ^d^ Zhang and Qu [72] used the same sample of Weibo users (*n* = 28) as a resting-state fMRI study by Zhang and Mo [67].

## 4. Discussion

The present article sought to systematically review existing research employing MRI methods to investigate the neural underpinnings of SNS use. In general, MRI studies investigating ‘SNS addiction’ are relatively scarce and a larger number of MRI studies have investigated either internet gaming or non-specific internet use addiction (e.g., 44 MRI studies investigating internet gaming disorder were identified in a recent systematic review; [87]). Below, we evaluate the main findings in relation to the key brain regions implicated in SNS use/addiction.

### 4.1. Ventral Striatum/Nucleus Accumbens

The ventral striatum, and in particular the NAc within the ventral striatum, appears to be a key region involved in the use of SNSs. The NAc is known to play a central role in the brain’s ‘reward circuit’ and is responsible for regulating motivation for drug-seeking behaviour in addiction [88]. Neuroimaging studies of substance use addictions have consistently shown greater ventral striatum/NAc activity in addicts presented with addiction-related cues [89,90] and there is also evidence that NAc activity is correlated with increased cravings in problem gamblers [21]. Therefore, MRI studies investigating SNS use might reasonably expect neuroadaptations of the ventral striatum/NAc to be associated with excessive/problematic SNS use or responses to SNS cues. Indeed, there is evidence that problematic SNS users show heightened activity in the ventral striatum when responding to SNS cues [63] and even regular users show greater activity in this region when performing tasks that simulate SNS use especially when the task involves cues related to social reward (e.g., ‘likes’) [73,74,75]. Furthermore, higher SNS engagement may also be characterised by reduced functional connectivity between the ventral striatum and DLPFC (reflecting reduced control) but increased connectivity in the ventral striatum-ventral medial PFC ‘reward pathway’ [37]. Multiple structural MRI studies have also found associations between more excessive/problematic SNS use and reduced ventral striatum volume. Montag et al. [46] provides the most compelling evidence for reduced NAc GMV in more excessive SNS users, since the study used a comparatively large sample size and employed an objective measure of SNS usage. Such a finding is also consistent with the substance addiction literature where reduced volume of structures within the brain’s reward system are typically observed in addicted individuals [91,92]. More excessive/problematic SNS use therefore seems to be characterised by reduced ventral striatum volume but heightened activity in this region in response to SNS cues or social rewards related to SNS use.

### 4.2. Prefrontal Cortex

The prefrontal cortex can be subdivided into three broad structures that comprise the lateral, medial and orbitofrontal regions. The lateral prefrontal regions are thought to be responsible for implementing cognitive control and executive functions such as working memory, selective attention and planning, while the medial prefrontal regions play a role in motivation, decision making and self-referential processing [93]. Due to its many connections with the limbic system the orbitofrontal cortex plays a vital role in emotional behaviour and reward processes and is responsible for forming reward expectations as well as representing reward value and subjective pleasantness [94]. The functions of the PFC are thus clearly relevant to the development of addiction and various structural and functional abnormalities of prefrontal regions have been documented in the substance use addiction literature [95]. Such abnormalities are thought to be responsible for the impaired inhibitory control and increased cravings/impulsivity experienced during addiction. Multiple studies identified in the present review have also implicated prefrontal regions in the use and problematic use of SNSs.

Nonetheless, evidence of structural PFC abnormalities being associated with SNS use is scarce. It is well established that drugs of abuse are associated with grey matter atrophy in the PFC [96,97,98] and there is also evidence that behavioural addictions such as gambling disorder are related to reduced cortical thickness in frontal regions [99]. Yet, only one study has reported such a relationship (reduced GMV of the right orbitofrontal cortex) in problematic SNS users [35]. However, this study did employ a large sample size when compared to the other structural MRI studies identified in this review. Notably, one study found that adolescents with more excessive SNS use showed higher cortical thickness in the lateral and medial PFC [56]. This finding is not entirely inconsistent with other addictive disorders since increased GMV in the right prefrontal cortex has also been observed in problem gamblers and is suggested to be a neuroadaptation in response to the increased cognitive demands required to control gambling impulses [100]. However, in their longitudinal study, Achterberg et al. [56] also reported accelerated cortical thinning in the lateral PFC in excessive SNS users, although this finding did not survive FDR correction. Thus, to date there is only weak evidence to suggest that SNS use is associated with structural changes in the PFC.

A larger number of studies have reported functional alterations of the PFC at rest and during task performance. The OFC has been implicated in only a few functional MRI studies, none of which correlated activity with excessive or problematic SNS use. Instead, increased functional connectivity between the OFC and central precuneus appears to be associated with sharing personal information online [64], while stronger connections to the dorsomedial PFC and temporal parietal junction facilitates SNS reposting [67]. Sherman et al. [76] also reported increased OFC activity when participants ‘liked’ another’s Instagram post. Thus, while abnormal OFC activity/connectivity has not been associated with more problematic SNS use, this region does seem to play an important role in active SNS engagement (e.g., posting, sharing and ‘liking’ SNS content).

The lateral prefrontal cortex (particularly the dorsolateral prefrontal cortex; DLPFC) has also been shown to be involved in SNS use behaviours. It is likely that the DLPFC is recruited to manage the cognitive and emotional demands of sharing personal and emotionally valenced content online [64,72]. Given the key role the DLPFC plays in implementing cognitive control [50], it is also unsurprising that more efficient connectivity with the ventral striatum is associated with lower SNS engagement (improved control) [37] while reduced connectivity with the dorsal attention network is associated with more problematic use (reduced control) [36]. Limited inhibitory control abilities in more problematic SNS users may also be reflected by reduced white matter integrity in the forceps minor (a white matter bundle connecting the lateral and medial surfaces of the frontal lobes) [57]. However, contrary to what might be expected, fMRI studies employing tasks of executive function have reported no abnormalities in PFC activation for problematic SNS users [38,63].

Finally, the medial PFC has been implicated in a range of fMRI studies. Increased functional connectivity between the medial PFC and ventral striatum in more excessive SNS users [37] and increased medial PFC activity in response to viewing SNS photos with many ‘likes’ [74,75] could potentially reflect an increased motivation to pursue SNS rewards. However, more problematic SNS users have been found to show reduced medial PFC activity when viewing cues depicting risky behaviours which might indicate impaired decision making abilities [77]. Other studies have also supported a key role for the medial PFC in the self-referential cognition network. Reduced medial PFC activity when making self-reflections might indicate deficits in self-referential cognition as a result of excessive SNS use [82]. Although, one study found increased medial PFC activity during self-judgements in more excessive users, which had no relationship with long-term negative outcomes [83]. Increased medial PFC activity when viewing personalised SNS videos [80] or when receiving negative SNS comments [79] may also reflect the processing of content relevant to the self.

### 4.3. Amygdala

The amygdala is another key structure implicated in the development of substance use addictions. The amygdala is engaged in emotional and motivational processes and forms a crucial component of the brain’s arousal/stress systems which are thought to play a key role in facilitating the transition to drug dependence and maintenance [101]. Meta-analyses of drug cue-reactivity studies have found cue-induced amygdala activity to be one of the most robust findings in the substance addiction literature [90,102], and rodent models have also shown that activating the amygdala intensifies motivation for drug consumption [103]. By contrast, no existing research has found an association between amygdala activity and problematic SNS use (although cue-reactivity studies are limited). Nonetheless, Sherman et al. [76] did find increased amygdala activity in regular SNS users when ‘liking’ another’s post, which is consistent with the amygdala’s role in motivation and reward expectancy (‘liking’ another’s post may in turn increase the likelihood of an individual reciprocating ‘likes’ on a future post, intensifying reward expectancy). Structurally, two studies reported an association between reduced amygdala volume and more excessive/problematic SNS use [47,49]. This finding is in keeping with observations in substance use disorders [52,104,105,106], where reduced amygdala volume is suggested to be responsible for increased susceptibility to emotion dysregulation, resulting in a reduced ability to manage cravings. Thus, in SNS use, the amygdala appears to play a role in decisions to distribute positive feedback to others, while reduced amygdala size may be predictive of more intense or harmful SNS use.

### 4.4. Cingulate Cortex

The cingulate cortex, principally the ACC (a central node of the cognitive control network; 50) but also the posterior cingulate cortex (PCC), has been widely implicated in substance use addictions [90,107,108]. Structural MRI studies have also identified reduced ACC volume in both substance use [109,110] and behavioural addictions (e.g., internet gaming disorder; [111]) reflecting diminished cognitive control abilities. In the present review, two studies were identified which found significant correlations between ACC volume and problematic SNS use. Montag et al. [48] found reduced subgenual ACC volume in more addicted SNS users, whereas He, Turel and Bechara [49] found an association between increased dorsal ACC volume and higher addiction severity. While this might indicate specific functions for ACC subregions in addiction, the finding of increased dorsal ACC volume in more problematic SNS users is still not consistent with the wider addiction literature. Indeed, neural deficits in the dorsal ACC have been proposed to constitute a hallmark neurocognitive deficit underlying addictive disorders [112] and stimulating the rostrodorsal ACC can help suppress alcohol cravings in individuals with alcohol use disorder [113]. In line with this, Sherman et al. [74] found that teenaged Instagram users showed deactivations of regions in the cognitive control network (including the dorsal ACC), when viewing Instagram posts depicting risky (vs. neutral) behaviours ostensibly posted by peers, which suggests disinhibited cognitive control in response to highly salient SNS cues. Although this effect appears to be age-specific [75], potentially reflecting an immature capacity for cognitive control during adolescence. The finding of increased ACC activity when ‘liking’ a peers Instagram post [76] also dovetails with evidence of an integral role for the ACC in linking motivational outcomes to behaviour [114]. Nonetheless, in contrast to what was hypothesised, neither of the two fMRI studies employing cognitive control tasks observed alterations of ACC activity in problematic SNS users [38,63], which is therefore inconsistent with similar studies investigating substance use [115] and other behavioural addictions [116].

The PCC constitutes a central structure of the default mode network (DMN), a neural network that is maximally activated at rest [117,118]. The DMN has been found to show increased activation and altered functional connectivity with various other regions (including regions in the dorsal and ventral attention networks) when viewing personalised TikTok videos and after reading SNS microblogs [68,80,81]. Such findings imply that passively consuming SNS content (particularly personalised content) may activate a neural state similar to that when mind wandering, which may facilitate prolonged use through modulating attention and inducing absent-minded scrolling/browsing of SNS feeds. Modifications of DMN functional connectivity have also been observed in substance use addictions which are proposed to facilitate cravings and relapse [119]. The DMN is thought to be predominantly engaged during phases of withdrawal and preoccupation in addiction and is thus active at the expense of the cognitive control network, limiting its capacity to manage cravings and prevent relapse.

### 4.5. Precuneus

The precuneus, a region which neighbours the PCC, is another core hub of the DMN and is therefore also implicated in studies demonstrating alterations of DMN activity and functional connectivity when viewing SNS content [68,80,81]. The precuneus is also thought to play an important role in a range of complex tasks including visuo-spatial imagery, memory retrieval and self-referential cognition [120]. As previously discussed, enhanced coupling between the precuneus and prefrontal regions appears to be associated with increased sharing of personal information on SNSs, whereas increased connectivity between the precuneus and anterior temporal pole (ATP) is negatively associated with self-related sharing [64]. The findings are therefore consistent with a role for the precuneus in self-referential cognition since enhanced connectivity with prefrontal regions is necessary to hold self-related information in working memory. In contrast, the ATP is engaged when thinking about the mental states of others [121], and thus thinking about how others might react to a post may inhibit frequent self-disclosures on SNSs. Other research has shown that the precuneus is also engaged during decisions to repost emotionally valenced messages online [72,79] and when viewing posts with many (vs. few) likes [74], suggesting that these SNS behaviours trigger self-relevance processing. As reviewed above, only one study implicated the precuneus in problematic SNS use during exposure to Instagram photos, suggesting a role for this region in cue reactivity and the transmission of visual information to motivational systems [77]. Meta-analyses have also found that cue-induced precuneus activity is associated with substance use [23,122] and behavioural addictions [123].

### 4.6. Temporal Parietal Junction

One function of the temporal parietal junction (TPJ) is to form a key node of the mentalising network, which enables cognitive empathy and perspective taking, allowing us to interpret the mental states of others [124]. Given that mentalising (or other functions of the TPJ) are not directly implicated in substance use or addictive behaviours, it is unsurprising that TPJ activation is rarely reported in addiction research. One study has linked reduced amygdala-TPJ connectivity to neuroticism, a personality trait which can influence addiction severity [125]. Nonetheless, the TPJ may be more relevant in the development of behavioural addictions that involve social communication and interpreting another’s mental state (e.g., SNS use). Increased grey matter density of the TPJ has been shown to be positively correlated with internet addiction [126], yet the only structural MRI study identified in the present review to implicate the TPJ found a stronger decrease in the surface area of this region in more excessive adolescent SNS users over a period of three years [56]. However, such disparities may reflect unique age-related differences between measures of grey matter morphology [127]. Furthermore, while Achterberg et al. [56] argued that stronger reductions in the surface area of the TPJ in more excessive SNS users was consistent with their hypothesis that SNS use would lead to accelerated maturation of brain regions important for social processing, they also noted that the correlation was weak and did not survive a correction for multiple tests. As reviewed above, the TPJ has also been implicated in regular (non-addicted) SNS reposting behaviours [67,72]. Thus, when reposting SNS messages (especially emotionally valenced messages) the TPJ may play an important role in enabling an understanding of how such messages will be viewed from another’s perspective, while excessive SNS use (at least during adolescence) may accelerate cortical thinning in this region, potentially contributing to a lack of impulse control [128].

### 4.7. Insula

A single structural MRI study has implicated the insula in problematic SNS use [54], indicating that grey matter deficits in this region may result in increased impulsivity and a preference for immediate gains in problematic users. This is consistent with the role of the insula in decision making and delaying gratification. Grey matter deficits of the insula have also been proposed to constitute an important structural marker of drug addictions. For example, in one study, both cocaine and heroin dependent patients were shown to have reduced grey matter in the posterior insula when compared to healthy controls [129]. The importance of the insula for addiction processes is further supported by evidence that damage to this region can cause cigarette smokers to spontaneously quit without relapse [130] and insular reactivity to addiction-related cues can predict relapse [131]. This is because the insula is thought to be responsible for forming conscious representations of the homeostatic imbalance caused by withdrawal which manifests as urges or cravings for drug consumption [55]. Despite its important role in the maintenance of substance use addictions, research is yet to associate functional alterations of the insula with more problematic SNS use. Nonetheless, as reviewed above, in regular users, insula activity and its connectivity with the TPJ has been associated with reposting emotionally valenced messages, ‘liking’ SNS photos and receiving valenced SNS comments [67,72,76,79]. Such patterns of activity are also consistent with a role for the insula in detecting salient stimuli and modulating emotional responses [132].

### 4.8. Other Observations

As well as identifying brain regions associated with SNS use, the present review also highlights methodological issues that future research should address. Firstly, seven different scales were employed across the identified articles to assess problematic/compulsive SNS use. The inclusion of certain diagnostic criteria assessed in some of these scales (e.g., escape/mood management criterion) have also been criticised for lacking clinical validity and pathologising normal use motives [133,134]. Thus, there is a need to establish consensus regarding the symptomatology of problematic SNS use and for diagnostic criteria to be assessed consistently across studies in order to improve comparability. Furthermore, the majority of studies that assess excessive SNS use have relied on self-reports of use intensity. Recent evidence suggests that individuals are often inaccurate in reporting the duration of their SNS use, displaying a tendency to overestimate their usage intensity [135,136]. Future research should aim to utilize objective measures of SNS use to ensure that excessive use behaviours are reliably assessed. Moreover, existing neuroimaging research is limited with large heterogeneity among studies. As such more MRI studies employing more consistent methods are required to establish firm conclusions about the neural underpinnings of excessive/problematic SNS use.

Additionally, the majority of research to date has focused on investigating the neural correlates of SNS use in adolescents and young adults. While this seems reasonable given that younger populations have increased exposure to SNSs and are thought to be more vulnerable to experiencing negative consequences [137], future research may also wish to assess whether excessive/problematic SNS use in older adults is associated with similar brain changes. Furthermore, many of the studies that implicate regions of the cognitive control network in the use of SNSs have inferred that deficits to these regions may be responsible for the development of more problematic or compulsive SNS use. Yet, neither of the two task-based fMRI studies employing cognitive control tasks have found evidence of dysfunctional activity within the cognitive control network in more problematic users [38,63]. This lack of evidence is surprising since impaired activity of the cognitive control network during tasks of executive functions has been consistently observed in individuals with substance use disorders and other behavioural addictions [112,138]. Thus, more task-based fMRI studies employing behavioural paradigms (e.g., Go/No-Go, Stroop, and Approach/Avoidance) are needed to better understand the cognitive/motivational processes underpinning SNS use and overuse. Moreover, longitudinal research is scarce and existing studies report mixed results. While only subtle differences in brain morphology have been attributed to excessive SNS use [56], some studies have shown widespread functional connectivity changes as a result of increased SNS use [70]. Yet, other research suggests that abnormal neural activity associated with increased SNS use has no long-term effects on clinical symptoms [83]. As such, it cannot yet be concluded that excessive/problematic SNS use is responsible for significant neuroadaptations that represent a disease process, as is observed in substance use disorders. Alternatively pre-existing neural abnormalities may be responsible for facilitating the development of more compulsive or problematic forms of SNS use.

While our review does not directly inform the treatment of problematic SNS use, our findings highlight the important role of (social) reward and its associated brain structures in driving this behaviour. Incorporating elements into intervention packages that focus on controlled and sensible seeking of SNS rewards (e.g., through limiting exposure to SNS notifications or disabling the ‘like’ function) may thus constitute a viable avenue to tackle problematic SNS use.

## 5. Conclusions

In sum, our systematic review of MRI studies investigating SNS use suggests that more excessive/problematic use does share some important neural similarities to substance abuse. In particular, the existing evidence leads us to conclude that problematic use may be characterised by: (1) reduced GMV of the ventral striatum, amygdala, subgenual anterior cingulate cortex, orbitofrontal cortex and posterior insula; (2) increased ventral striatum and precuneus activity in response to SNS cues; (3) abnormal functional connectivity between middle occipital gyrus and DLPFC and the dorsal attention network; (4) impaired white matter integrity in the corpus callosum and forceps minor. In regular users, regions including the temporal parietal junction, ventral striatum, precuneus, insula and frontal lobes have been implicated in active SNS engagement (i.e., sharing/reposting/‘liking’ SNS content), while altered functional connectivity of the default mode network is associated with passive consumption of SNS content (particularly personalised recommended videos). Nonetheless, given that these regions are not consistently implicated across studies, and considering the large heterogeneity of current studies (e.g., in the type of SNS under investigation, the scales used to assess SNS use/addiction, and the experimental tasks employed), our conclusions should remain tentative. To date, the majority of research investigating the neural correlates of problematic/compulsive SNS use are structural MRI studies, and thus more fMRI research is required to elucidate the functional alterations that might underpin a ‘SNS addiction’. Furthermore, most existing neuroimaging research is limited by small sample sizes, recruiting from student populations, and employing self-reports of usage intensity. More large-scale and longitudinal research employing objective measures of SNS use are essential to establish stronger conclusions regarding the neurobiological effects of SNSs.

## Data Availability

Not applicable to this article as no new data were created or analysed. The supporting information can be found in Supplementary Materials.

## Supplementary Materials

The following supporting information can be downloaded at: https://www.mdpi.com/2076-3425/13/5/787/s1, Table S1: Quality assessment ratings for structural MRI studies adapted from EPHPP criteria; Table S2. Quality assessment ratings for resting state fMRI studies adapted from EPHPP criteria; Table S3. Quality assessment ratings for task-based fMRI studies adapted from EPHPP criteria.
