# Correction: Wadsley, M.; Ihssen, N. A Systematic Review of Structural and Functional MRI Studies Investigating Social Networking Site Use. *Brain Sci.* 2023, *13*, 787

**DOI:** 10.3390/brainsci13071079

**Published:** 2023-07-17

**Authors:** Michael Wadsley, Niklas Ihssen

**Affiliations:** Department of Psychology, Durham University, Durham DH1 3LE, UK; niklas.ihssen@durham.ac.uk

## Citation Correction

During the final proofreading step of this paper [1], an error in the citation order was introduced, which affected the citation order from references [63] to [85] and the respective callouts in the main text. The changes are as follows:

Last paragraph of Section 3.2, “In another study by the same group and recruiting from the same pool of participants, Hu et al. [69] used a longitudinal design to investigate changes in functional connectivity after a month of excessive SNS use.” The reference number is now [70].

7th paragraph of Section 3.3, “Analysing data from the same sample as a previous resting-state fMRI study [66].” The reference number is [67]. 

8th paragraph of Section 3.3, “that were extracted from their own account as well as six generalised recommended videos for new users [79].” and “In an extension of this work, the same group published further analyses of the same dataset using graph theory to investigate the functional connectivity between seven networks [80].”. The reference numbers are [80] and [81], respectively. 

Last paragraph of Section 3.3, “In a study by Leménager et al. [81],” and “A more recent study used a similar self-concept paradigm in which participants had to make self-judgements on academic, physical and prosocial traits from their own perspective and from the perspective of others [82].” The reference numbers are now [82] and [83], respectively. 

In Table 2, the reference number Hu, Cui et al. [69] is now [68].

In Table 3, the references to Meshi, Morawetz and Heekeren (2013) [72], Sherman et al. (2016) [73], Sherman, Greenfield et al. (2018) [74], Sherman, Hernandez et al. (2018) [75] are incorrect (references appear in the author, sample, and design columns). References numbers are now [73], [74], [75], and [76], respectively.

In Section 4.1, “Indeed, there is evidence that problematic SNS users show heightened activity in the ventral striatum when responding to SNS cues [71].” The reference number is now [63].

In Section 4.1, “and even regular users show greater activity in this region when performing tasks that simulate SNS use especially when the task involves cues related to social reward (e.g., ‘likes’) [72–74].” References number are now [73–75], respectively.

In 3rd paragraph of Section 4.2, “Sherman et al. [75] also reported increased OFC activity when participants ‘liked’ another’s Instagram post.” The reference number is [76].

In 4th paragraph of Section 4.2, “It is likely that the DLPFC is recruited to manage the cognitive and emotional demands of sharing personal and emotionally valenced content online [63,79].” and “However, contrary to what might be expected, fMRI studies employing tasks of executive function have reported no abnormalities in PFC activation for problematic SNS users [38,71].” The references numbers are now [64,72] and [38,63], respectively. 

Last paragraph of Section 4.2, “increased medial PFC activity in response to viewing SNS photos with many ‘likes’ [73,74]” and “However, more problematic SNS users have been found to show reduced medial PFC activity when viewing cues depicting risky behaviours which might indicate impaired decision making abilities [76].” The reference number are now [74,75] and [77], respectively.

Last paragraph of Section 4.2, “or when receiving negative SNS comments [78] may also reflect the processing of content relevant to the self.” The reference number is [79]. 

In Section 4.3, “Nonetheless, Sherman et al. [75] did find increased amygdala activity in regular SNS users when ‘liking’ another’s post”. The reference number is [76].

First paragraph of Section 4.4, “In line with this, Sherman et al. [73] found that teenaged Instagram users showed deactivations of regions in the cognitive control network”, “Although this effect appears to be age-specific [74]”, and “The finding of increased ACC activity when ‘liking’ a peers Instagram post [75]”. The reference numbers are now [74], [75], and [76], respectively.

In Section 4.5, “Other research has shown that the precuneus is also engaged during decisions to repost emotionally valenced messages online [78,79]”. The reference numbers are now [72,79].

In Section 4.5, “and when viewing posts with many (vs. few) likes [73]”. The reference number is now [74].

In Section 4.5, “As reviewed above, only one study implicated the precuneus in problematic SNS use during exposure to Instagram photos, suggesting a role for this region in cue reactivity and the transmission of visual information to motivational systems [76]”. The reference number is now [77].

In Section 4.6, “As reviewed above, the TPJ has also been implicated in regular (non-addicted) SNS reposting behaviours [66,79].” The reference numbers are now [67,72].

In Section 4.7, “Nonetheless, as reviewed above, in regular users, insula activity and its connectivity with the TPJ has been associated with reposting emotionally valenced messages, ‘liking’ SNS photos and receiving valenced SNS comments [67,75,78,79]”. The reference numbers are now [67,72,76,79].

## References

The authors would like to reorder references [83–85] in the References list. The correct order should be the following:

83.Peters, S.; Van der Cruijsen, R.; van der Aar, L.; Spaans, J.; Becht, A.; Crone, E. Social media use and the not-so-imaginary audience: Behavioral and neural mechanisms underlying the influence on self-concept. *Dev. Cogn. Neurosci.*
**2021**, *48*, 100921.84.Wölfling, K.; Beutel, M.; Müller, K. Construction of a standardized clinical interview to assess internet addiction: First findings regarding the usefulness of AICA-C. *J. Addict. Res. Ther.*
**2012**, *6*, 003.85.Ellison, N.B.; Steinfield, C.; Lampe, C. The benefits of Facebook “friends:” Social capital and college students’ use of online social network sites. *J. Comput.-Mediat. Commun.*
**2007**, *12*, 1143–1168.

The references list and corresponding citations throughout the manuscript have been revised.

The authors state that the scientific conclusions are unaffected. This correction was approved by the Academic Editor. The original publication has also been updated.
