# Supplementary material for: A Systematic Review of Structural and Functional MRI Studies Investigating Social Networking Site Use
Source: Brain Sci. 2023 May 11;13(5):787. doi: 10.3390/brainsci13050787 (PMC10216498; doi:10.3390/brainsci13050787)
Supplement: Supplementary file 1 [file brainsci-13-00787-s001.zip › brainsci-2305382-supplementary.pdf]

Supplementary Table S1. Quality assessment ratings for structural MRI studies adapted from EPHPP criteria

	Achterberg et al. (2022)	He, Turel and Bechara (2017)	He, Turel, Brevers, et al. (2017)	He et al. (2018)	Lee et al. (2019)	Montag et al. (2017)	Montag et al. (2018)	Turel et al. (2018a)	Turel et al. (2018b)
**A. Selection bias**									
1. Are the individuals selected to participate likely to be representative of the target population? (yes = 1; no/unclear = 0)	1	0	0	0	1	0	0	0	0
**B. Study design**									
1. Was sample size sufficient or derived from a power calculation? (yes = 1; no = 0)	1	0	1	0	1	1	1	1	1
2. Are inclusion and exclusion criteria clearly described? (yes = 1; no = 0)	1	1	1	1	1	1	1	1	1
3. Were appropriate details of sample characteristics included? (yes = 1; no = 0)	1	1	1	1	1	1	1	1	1
**C. Neuroimaging methodology**									
1. Is a validated method of data acquisition used? (yes = 1; no/unclear = 0)	1	1	1	1	1	1	1	1	1
2. Is a validated method of data processing used? (yes = 1; no/unclear = 0)	1	1	1	1	1	1	1	1	1
**D. Analysis**									
1. Was analysis hypothesis driven? (yes = 1; no/unclear = 0)	1	1	1	1	1	1	1	1	1
2. Has the study controlled for potential confounders? (yes = 1; no = 0)	1	1	1	1	1	1	1	1	1

3. Has the study adjusted for multiple comparisons if appropriate? (yes/ not applicable = 1; no/unclear = 0)	1	1	1	1	1	1	0	1	1
4. Are statistical methods appropriate for study design/sample size? (yes = 1; no/unclear = 0)	1	0	1	0	1	1	1	1	1
**Total score (0-10)**	10	7	9	7	10	9	8	9	9
**Global rating (High = 10-8; Moderate = 5-7; Low = 0-4)**	High	Moderat e	High	Moder ate	High	High	High	High	High

Supplementary Table S2. Quality assessment ratings for resting state fMRI studies adapted from EPHPP criteria

	Hu, Cui, et al. (2022)	Hu, Yu, et al. (2022)	Lee et al. (2021)	Meshi et al. (2016)	Wilmer et al. (2019)	Zhang and Mo (2016)
**A. Selection bias**						
1. Are the individuals selected to participate likely to be representative of the target population? (yes = 1; no/unclear = 0)	0	0	1	0	0	0
**B. Study design**						
1. Was sample size sufficient or derived from a power calculation? (yes = 1; no = 0)	0	0	1	1	0	0
2. Are inclusion and exclusion criteria clearly described? (yes = 1; no = 0)	1	1	1	1	1	1
3. Were appropriate details of sample characteristics included? (yes = 1; no = 0)	1	1	1	1	1	0
**C. Neuroimaging methodology**						
1. Is a validated method of data acquisition used? (yes = 1; no/unclear = 0)	1	1	1	1	1	1
2. Is a validated method of data processing used? (yes = 1; no/unclear = 0)	1	1	1	1	1	1
**D. Analysis**						
1. Was analysis hypothesis driven? (yes = 1; no/unclear = 0)	0	1	1	1	1	1
2. Has the study controlled for potential confounders? (yes = 1; no = 0)	1	1	1	1	1	0

3. Has the study adjusted for multiple comparisons if appropriate? (yes/ not applicable = 1; no/unclear = 0)	1	1	1	1	0	1
4. Are statistical methods appropriate for study design/sample size? (yes = 1; no/unclear = 0)	1	1	1	1	1	1
**Total score (0-10)**	7	8	10	9	7	6
**Global rating (High = 10-8; Moderate = 5-7; Low = 0-4)**	Moderate	High	High	High	Moderate	Moderate

Supplementary Table S3. Quality assessment ratings for task-based fMRI studies adapted from EPHPP criteria

	Dieter et al. (2017)	Leménager et al. (2016)	Meshi et al. (2013)	Nasser et al. (2020)	Peters et al. (2021)	Sherman et al. (2016)	Sherman, Greenfield, et al. (2018)	Sherman, Hernandez, et al. (2018)	Su, Zhou, Gong, et al. (2021)	Su, Zhou, Wang, et al. (2021)	Turel et al. (2014)	Wikman et al. (2022)	Zhang and Qu (2020)
**A. Selection bias**													
1. Are the individuals selected to participate likely to be representative of the target population? (yes = 1; no/unclear = 0)	1	1	0	0	1	1	1	0	0	0	0	1	0
**B. Study design**													
1. Was sample size sufficient or derived from a power calculation? (yes = 1; no = 0)	0	0	1	0	1	1	1	1	1	1	0	1	0
2. Are inclusion and exclusion criteria clearly described? (yes = 1; no = 0)	1	1	1	1	1	1	1	1	1	1	1	1	1
3. Were appropriate details of sample characteristics included? (yes = 1; no = 0)	1	1	1	1	1	0	0	0	0	0	1	1	0
**C. Neuroimaging methodology**													
1. Is a validated method of data acquisition used? (yes = 1; no/unclear = 0)	1	1	1	1	1	1	1	1	1	1	1	1	1
2. Is a validated method of data processing used? (yes = 1; no/unclear = 0)	1	1	1	1	1	1	1	1	1	1	1	1	1
**D. Analysis**													

1. Was analysis hypothesis driven? (yes = 1; no/unclear = 0)	1	1	1	1	1	1	1	1	1	1	1	1	1
2. Has the study controlled for potential confounders? (yes = 1; no = 0)	1	0	1	0	1	1	1	1	1	1	1	1	1
3. Has the study adjusted for multiple comparisons if appropriate? (yes/ not applicable = 1; no/unclear = 0)	1	1	1	1	1	1	1	1	1	1	1	1	1
4. Are statistical methods appropriate for study design/sample size? (yes = 1; no/unclear = 0)	0	0	1	0	1	1	1	1	1	1	0	1	1
**Total score (0-10)**	8	7	9	6	10	9	9	8	8	8	7	10	7
**Global rating (High = 10-8; Moderate = 5-7; Low = 0-4)**	High	Moderate	High	Moderate	High	High	High	High	High	High	Moderate	High	Moderate
